# Modern botanical analogue of endangered Yak (*Bos mutus*) dung from India: Plausible linkage with extant and extinct megaherbivores

**DOI:** 10.1371/journal.pone.0202723

**Published:** 2019-03-06

**Authors:** Sadhan K. Basumatary, Hukam Singh, H. Gregory McDonald, Swati Tripathi, Anil K. Pokharia

**Affiliations:** 1 Birbal Sahni Institute of Palaeosciences, Lucknow, Uttar Pradesh, India; 2 Bureau of Land Management, South, Salt Lake City, Utah, United States of America; Nanjing Institute of Geology and Palaeontology, Chinese Academy of Sciences, CHINA

## Abstract

The study reports the micro- and macrobotanical remains on wild Yak dung, providing evidence for understanding the diet, habitat, and ecology of extant and extinct megaherbivores. Grasses are the primary diet of the yak as indicated by the abundance of grass pollen and phytoliths. Other associated non-arboreal and arboreal taxa namely, Cyperacaeae, Rosaceae, Chenopodiaceae, *Artemisia*, *Prunus*, and *Rhododendron* are also important dietary plants for their living. The observation of plant macrobotanical remains especially the vegetative part and seeds of the grasses and Cyperaceae is also in agreement with the palynodata. The documented micro- and macrobotanical data are indicative of both Alpine meadow and steppe vegetation under cold and dry climate which exactly reflected the current vegetation composition and climate in the region. The recovery of *Botryococcus*, *Arcella*, and diatom was observed in trace amounts in the palynoassemblage which would have been incorporated in the dung through the ingestion of water and are indicative of the presence of perennial water system in the region. Energy dispersive spectroscopy analysis marked that the element contained in dung samples has variation in relation to the summer and winter, which might be due to the availability of the food plants and vegetation. This generated multiproxy data serves as a strong supplementary data for modern pollen and vegetation relationships based on surface soil samples in the region. The recorded multiproxy data could also be useful to interpret the relationship between the coprolites of herbivorous fauna and the palaeodietary, the palaeoecology in the region, and to correlate with other mega herbivores in a global context.

## Introduction

Recently, there has been an increasing interest in the study of pollen and non-pollen palynomorphs preserved in herbivore dung and how dung can serve as a substrate for their preservation [1–3]. This added information in better understanding the relationship between dietary habits of herbivores, the local vegetation composition and climate in a region. It also provides knowledge of herbivorous animals preferred diet which is dependent on the availability of diverse plant species in relation to their habitat. The diversity of available plants and their relative abundance, in turn, reflects the climate of the region. The study of the modern pollen deposition on the landscape forms a critical dataset and a prerequisite to understanding the palaeovegetation and climate in the region [4–8].

The systematic study of the relationship between modern pollen and vegetation in the higher parts of the Himalayan Mountains is very difficult due to hilly terrain and limited availability of soil samples. Consequently, the soil samples may not serve as a modern analogue that would permit an accurate interpretation of the palaeoecology in the region. Previously, some workers have only examined surface soil samples in order to understand the modern pollen and vegetation relationship in the higher Himalayan region [9,10]. A complementary data set can be provided by an examination of modern herbivore dung and which can also serve as a source of modern analogues of local and regional vegetation [1–3, 11, 12]. Additionally, studies of coprolite palynology, especially in North America, have also been used to interpret the prehistoric diet of both human beings and animals and to determine the local vegetation [13–15]. It is clear that pollen and spores incorporated into the stomach contents also reflect the composition of local vegetation and also reflects regional climate [16,17]. The study of the macrobotanical remains from a dung midden of an arctic ground squirrel and horse [17,18] provided information on both the animal’s diet and the local vegetation. The distribution of herbivorous animals within an ecosystem is often dependent on vegetation composition and its regional distribution [19–22]. Study of both diatoms and phytoliths in the dung can also serve as a powerful proxy for palaeoenvironmental reconstruction and recognition of the presence of domestic herbivores [23–28].

The main aim of this study is to document the presence of both the micro- and macrobotanical remains in wild Yak (*Bos mutus*) dung in order to determine the dietary preferences of the species and their relationship to the local vegetation composition and climate. Since large animals may play an important role in the biogeochemical cycle of the ecosystem [20,29,30], we have conducted FESEM-EDS analysis in this study in order to determine the relationship between the elements contained in Yak dung derived from the current vegetation composition and their dietary habits in the region.

## Study sites, vegetation and fauna

**Fig 1:**
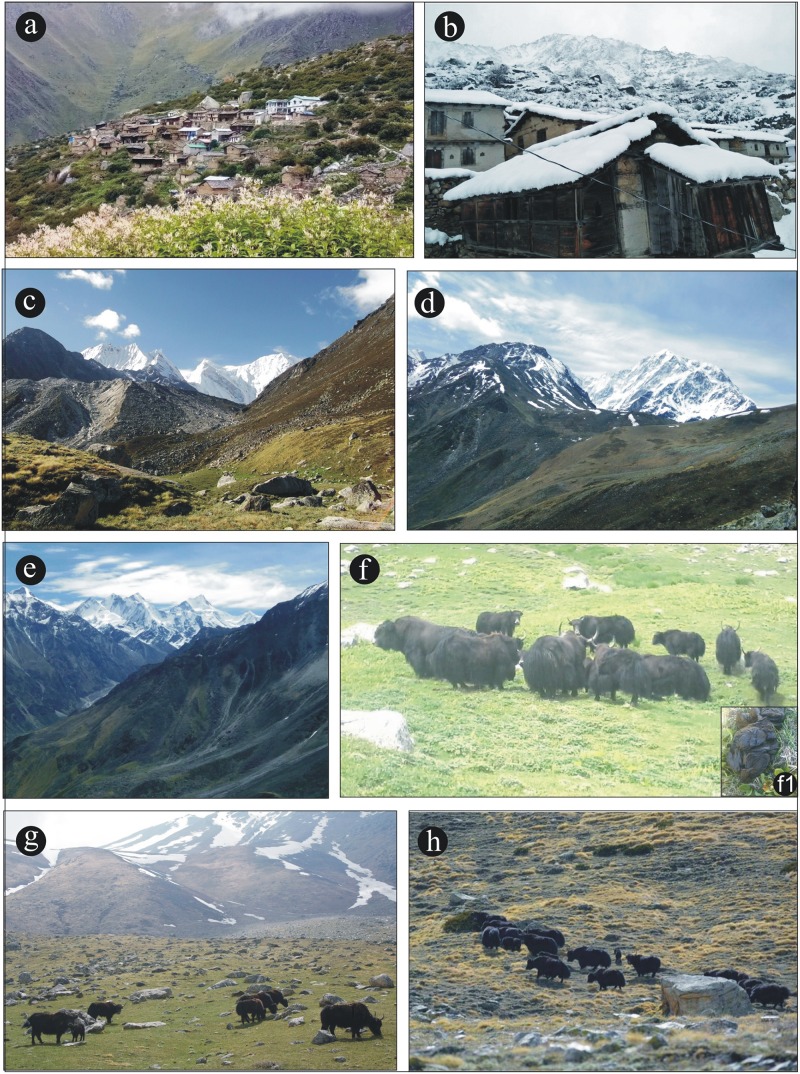
Field photographs. a. Dronagiri village inhabited by Bhotiyas near Indo-Tibet border, b. A view during winter snow fall, c. A view of Alpine meadow vegetation, d, & e. A view of Alpine steppe vegetation, f. A group of wild Yak during resting time in Alpine meadow vegetation, f1. Close up of Yak dung, g. A group of yak during grazing in Alpine steppe vegetation surrounding glacier point, h. Yak migration toward higher altitude during summer.

**Fig 2:**
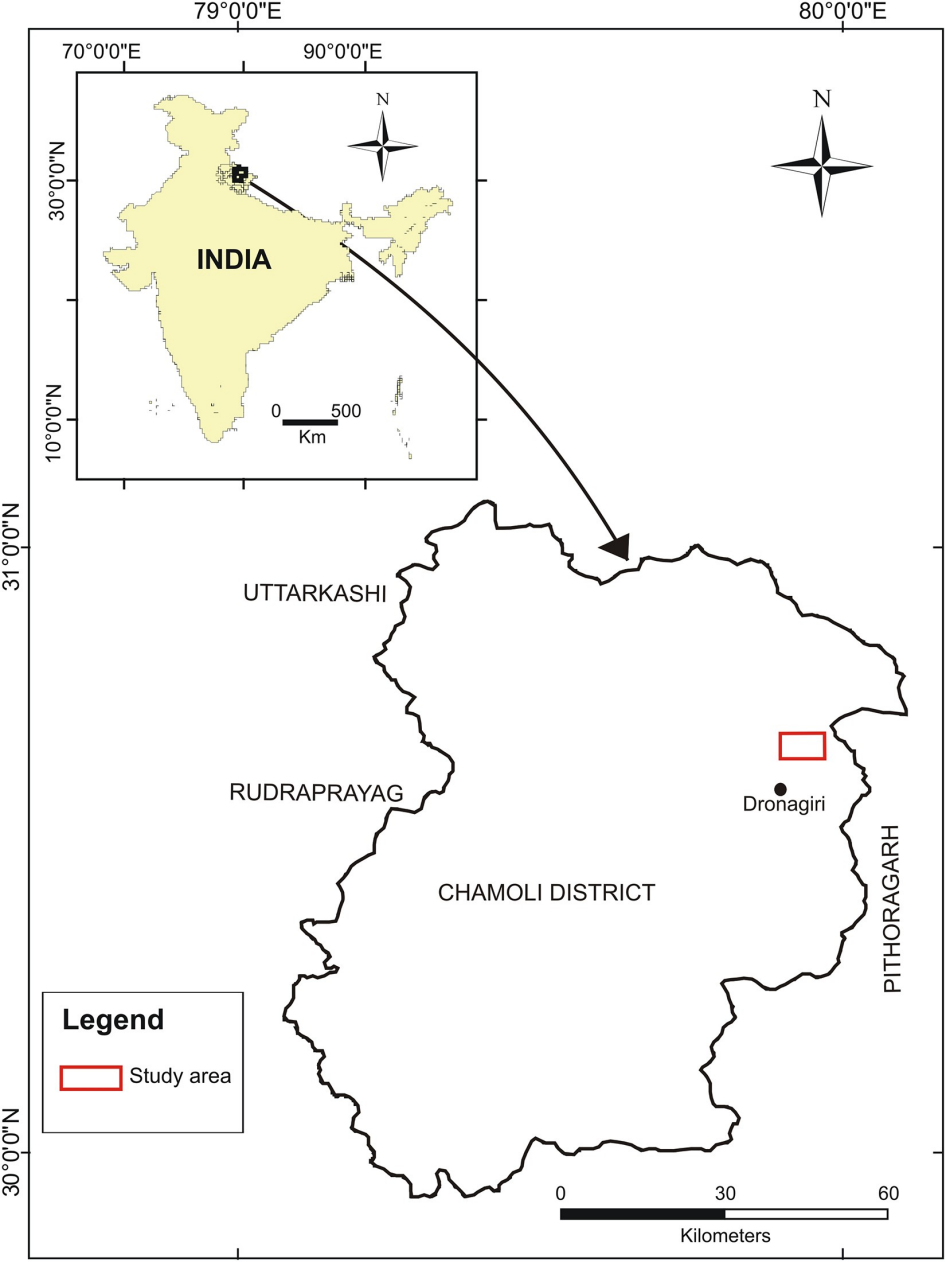
Map showing the location of the studied area.

The distribution of the wild Yak in India is very restricted and confined to higher elevations of the Himalaya. For this study we have selected a region, 30°38' 27.61''N to 30° 40' 47.20''N and 79°54' 16.38''E to 79°56' 35.58''E, which is around 12 km north from the Dronagiri village areas (Fig 1a and 1b) in the Chamoli district of Uttrakhand (India) based on the availability of the wild Yak and accessible terrain (Fig 2).

Vegetation in the studied area consists of Alpine meadow at lower elevations at 3300 meters above sea level (asl) and Alpine steppe at higher altitudes above 4000 meters asl (Fig 1c, 1d and 1e). Alpine meadow vegetation is mainly composed members of the Poaceae, Cyperaceae, and Asteraceae. Other associated taxa include members of the Polygonaceae, Onagraceae, Liliaceae, Rosaceae, Balsaminaceae, Chenopodiaceae, Amaranthaceae, and Ranunculaceae which grow luxuriantly in the region along with scattered shrubby elements, mainly consists of *Rhododendron campanulatum*, *Prunus armeniaca*, *Juniperus squamata*, *Juniperus indica*, and *Rosa macrophylla*. Ferns and their allies including *Equisetum diffusum*, *Pteridiumrevolutum*, *Adiantum venustrum*, *Asplenium fortanum*, and *Lycopodium selago* are also common.

Plants characteristic of the Alpine steppe include Poaceae, *Artemisia*, *Carex melanantha*, *Caragana*, *Stipa orientalis*, and *Lonicera* along with scattered shrubby elements namely, *Ephedra gerardiana*, *Haloxylon thomsonii*, and *Capparis spinosa* [31]. The Yak is one of the mega herbivores found in the higher Himalayan region of southern central Asia, the Tibetan Plateau and north Russia and Mongolia; it is considered to be critically endangered [32–34] (Fig 1f and 1g). The yak prefers habitats at high altitude, with a cool climate and generally it will tolerate temperatures as low as -40°C [35]. The climate of the region is mild during summer and very cold during winter. The maximum temperature ranges up to 12°C in summer and down to -20°C during winter. Other associated herbivorous mammals in the region include *Hemitragus jemlahicus* (Himalayan tahr) and *Moschus leucogaster* (White-bellied musk deer).

## Materials and methods

### Field work

The study area is a hilly terrain, which is open to all and there is no need of authority’s permission for the dung collection. In 2017, during the summer (March-July), the second author (HK) who is native of the region surveyed the site and collected11 fresh/semi-dry Yak dung samples based on their size **(**Fig 1f), each consisting of approximately 200g, from the different locations of the studied areas. Similarly, during winter (November-January), another 11 dung samples of similar size were also collected from the same areas. The collected dung samples were packed separately in polythene bags to avoid contamination before laboratory processing.

### Laboratory work

#### Macrobotanical analysis

For the macrobotanical analysis, 50 g of each dung sample both from summer and winter seasons were gently boiled in 200 ml 5% KOH solution. After boiling, the material was sieved through a 150 μm mesh. The material was washed for 2 to 4 times with distilled water and observed systematically under Stereobinocular (Leica Z6APO) microscope, and photographs were taken with a Leica DFC295 camera. Identifications were made through the consultation of published literature and the seeds and vegetative plant specimens preserve in BSIP herbarium.

#### Microbotanical analysis

The dung samples were processed for pollen using the standard acetolysis method [36]. Samples were successively treated with 10% aqueous potassium hydroxide (KOH) solution to deflocculate from the sediments, 40% hydrofluoric acid (HF) to dissolve silica, and acetolysis (9:1 anhydrous acetic anhydrite to concentrated sulphuric acid, (H_2_SO_4_) for the removal of cellulose. After that, the samples were treated twice with glacial acetic acid (GAA) and washed 3 or 4 times with distilled water. The samples were then transferred to a 50% glycerol solution with a few drops of phenol to protect them against microbial decomposition. Excluding the fungal spores, 218 to 280 pollen grains and fern spores were counted from each sample to produce the pollen spectra (S1 Table). The recovered pollen taxa were categorized as arboreal taxa (tree and shrub), non-arboreal taxa (marshy and terrestrial herb), and ferns. For the identification of pollen grains, we consulted the reference slides in the Birbal Sahni Institute of Palaeosciences (BSIP) herbarium in Lucknow (India) as well as published papers and photographs [37, 38].

For the diatom analysis, the samples were treated with concentrated hydrochloric acid (HCl) to dissolve carbonates and then treated with a mixture of hot nitric acid (HNO_3_) and potassium dichromate to dissolve organic materials. The samples were then washed with distilled water for 2 to 4 times and permanently mounted on a slide with Canada balsam for microscopic observation. The number of diatoms in the summer samples was very low and not suitable to make a proper diatom spectrum; no diatoms were observed in the winter samples. The phytoliths were observed on the same diatom slide because of the availability and clarity in the assemblage. Observation and microphotographs were done using an Olympus BX-61 microscope with DP-25 digital camera under 40x magnification. The identification of the phytoliths was based on the published literature [39].

#### Statistical analysis

The statistical significance of the quantified data of pollen frequency obtained from the dung samples was determined by SPSS 11.5.0, USA. A probability of *p*-value ≤ 0.05 was taken to indicate statistical significance. The resulting data were imported into Unscrambler X Software package (Version 10.0.1, CAMO, USA) for multivariate unsupervised PCA.

#### FESEM-EDS analysis

The Field Emission Scanning Electron Microscope (FESEM) with Energy Dispersive Spectroscopy (EDS) analysis was also performed using FESEM (JEOL, JSM-7610F) equipped with EDS (EDAX, USA instrument) operated at 25 keV to determine the elemental composition of the yak dung.

## Results

### Microbotanical assemblage from summer dung

**Fig 3:**
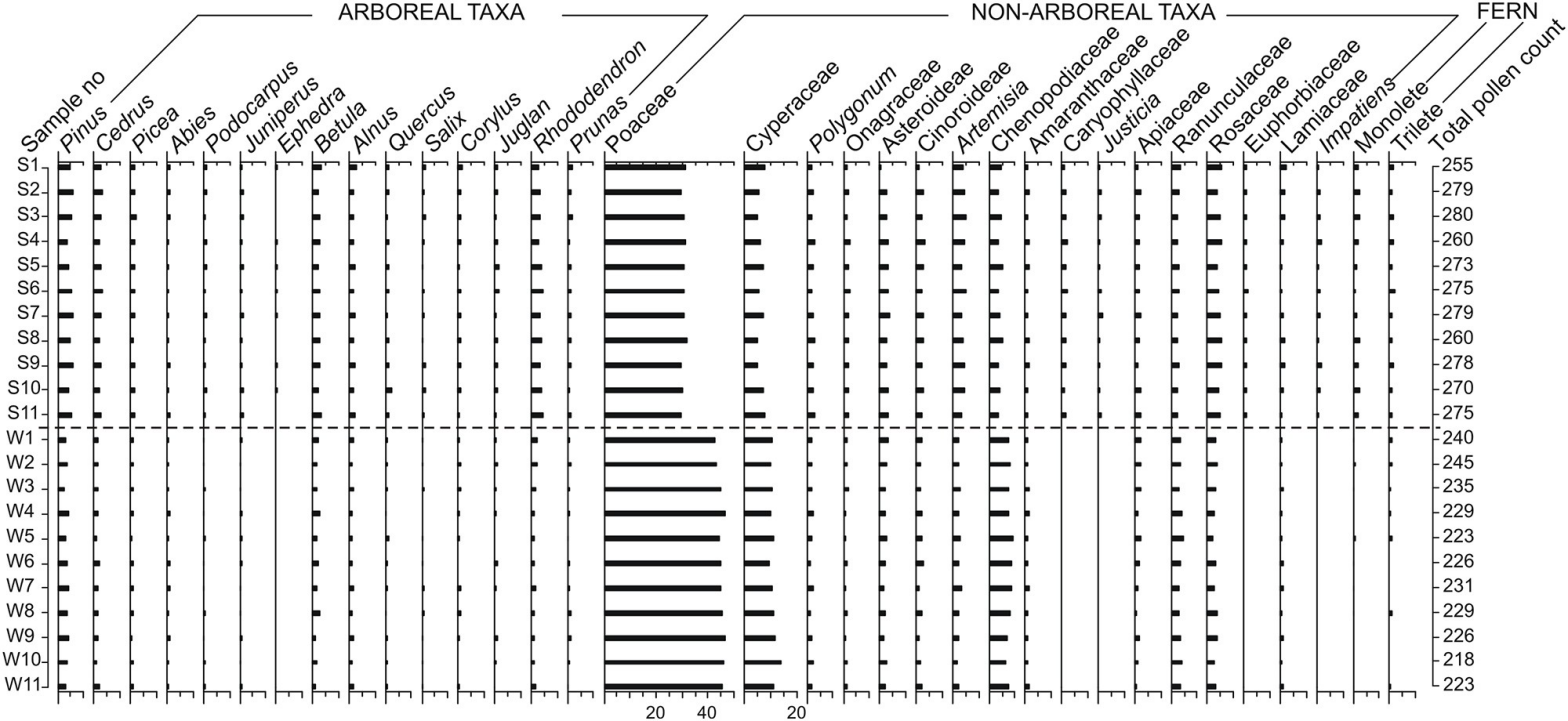
Pollen spectra of the studied Yak dung samples.

**Fig 4:**
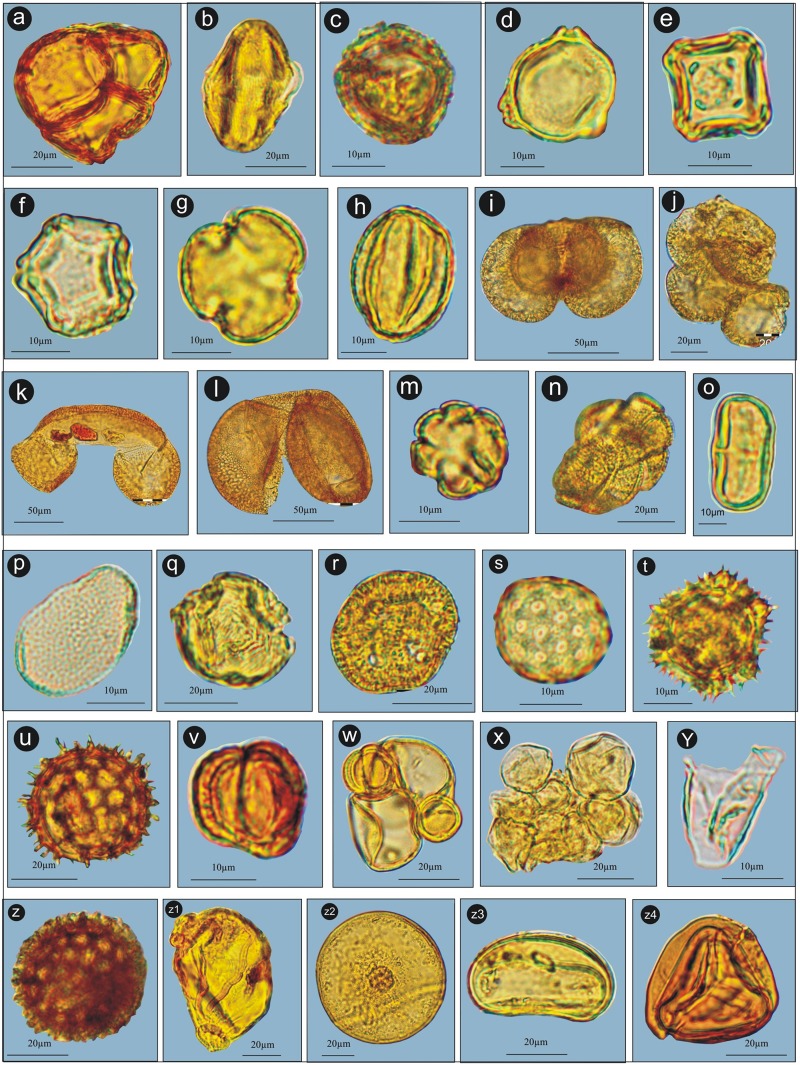
Palynoassemblage recovered from the Yak dung samples. a. *Rhododendron*, b. *Prunus*, c. *Juniperus*, d. *Betula*, e. *Alnus*, f. *Corylus*, g. *Salix*, h. *Quercus*, i. *Pinus*, j. *Pinus* pollen in clumping, k. *Abies*, l. *Cedrus*, m. Euphorbiaceae, n. Convolvulaceae, o. Apiaceae, p. *Impatiens*, q. Rosaceae, r. Amaranthaceae, s. Chenopodiaceae, t. Asteroideae, u. Cinoroideae, v. *Artemisia*, w. *Artemisia* pollen associated with Poaceae, x. Poaceae pollen in clumping, y. Cyperaceae, z. *Polygonum*, z2. Onagraceae, z1. *Arcella*, z3. Monolete, z4. Trilete.

The 11 dung samples (S1-S11) collected from the studied area were characterized by the dominance of non-arboreals (71.2%), over arboreals (25.8%). The ferns, both monolete and trilete, comprised 3.0%, of the sample. Among non-arboreal taxa, Poaceae was dominant (30.6%), followed by Cyperaceae (6.3%), and *Artemisia* (4.4%). Among arboreals the local taxa, *Rhododendron* and *Juniperus* were 3.5% and 0.9% respectively and the other extra-local arboreal taxa, *Pinus* and *Betula* were 5.0% and 2.6% (Figs 3 and 4).

**Fig 5:**
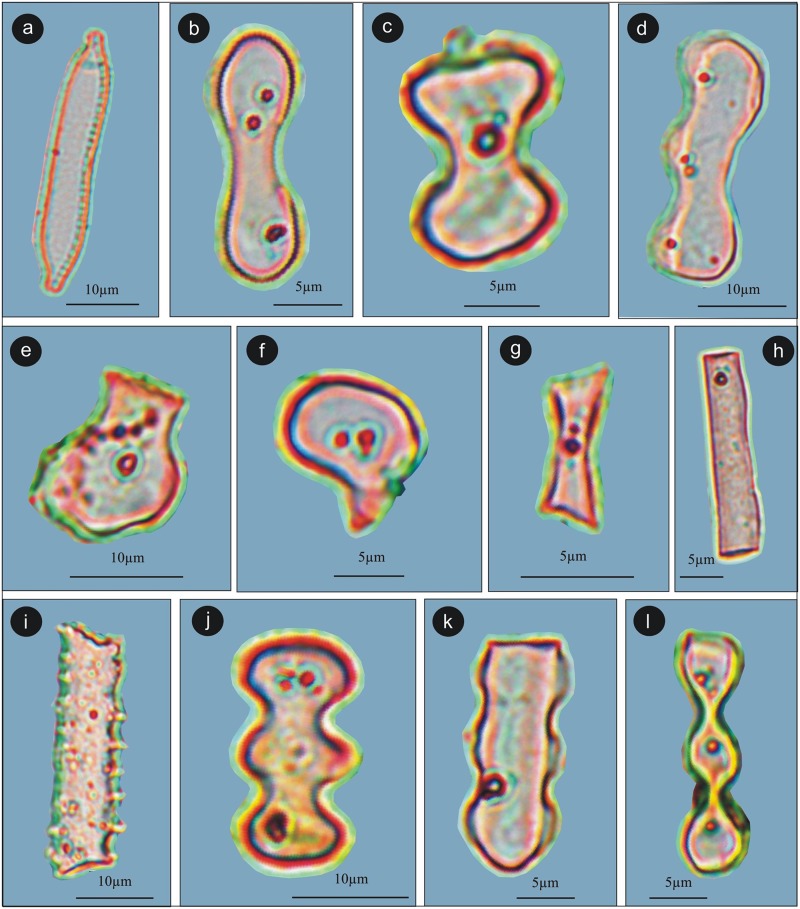
Diatom and phytoliths assemblage of the studied samples. a. *Hantzschia*, b. Dumbel bilobate, c. Bilobate short cell, d. Wavy, e. Cuneiform Bulliform cell, f. Bulliform, g. Rondel, h. Elongated smooth long cell, i. Elongated echinate long cell, j. Polylobate, k. Polylobate, l. Cylindrical polylobate.

Trace amounts of the diatom, *Hantzschia*, was present in all the samples. A variety of phytoliths were also observed in the same samples. Dumbbell bilobate morphology was dominant followed by elongated smooth long cell and bilobate. The others such as cuneiform, bulliform cell, rondel, and polylobate were also present in the assemblage (Fig 5).

### Microbotanical assemblage from winter dung

The 11 dung samples (W1-W11) collected from the study area are characterized by the dominance of non-arboreals (83.4%), over arboreals (15.8%). The ferns, both monolete and trilete, comprised 0.8%, of the sample. Among non-arboreal taxa, the Poaceae is dominant (45.0%), followed by Cyperaceae (11.0%), and Chenopodiaceae (7.8%). Among arboreals the local taxa, *Rhododendron* and *Juniperus* were represented at the value of 1.5% and 0.7% respectively. The other extra-local arboreal taxa, *Pinus*, and *Betula* were 3.5% and 1.9% respectively (Figs 3 and 4).

Diatoms are absent in all the studied samples. Phytoliths in the same samples are present with the dumbbell bilobate morphology dominant, as in the summer sample, followed by bilobate short cell and the elongated smooth long cell respectively. Other morphologies such as elongated echinate long cell, polylobate, and rondel are also regularly present in the assemblage. (Fig 5).

### PCA results

**Fig 6:**
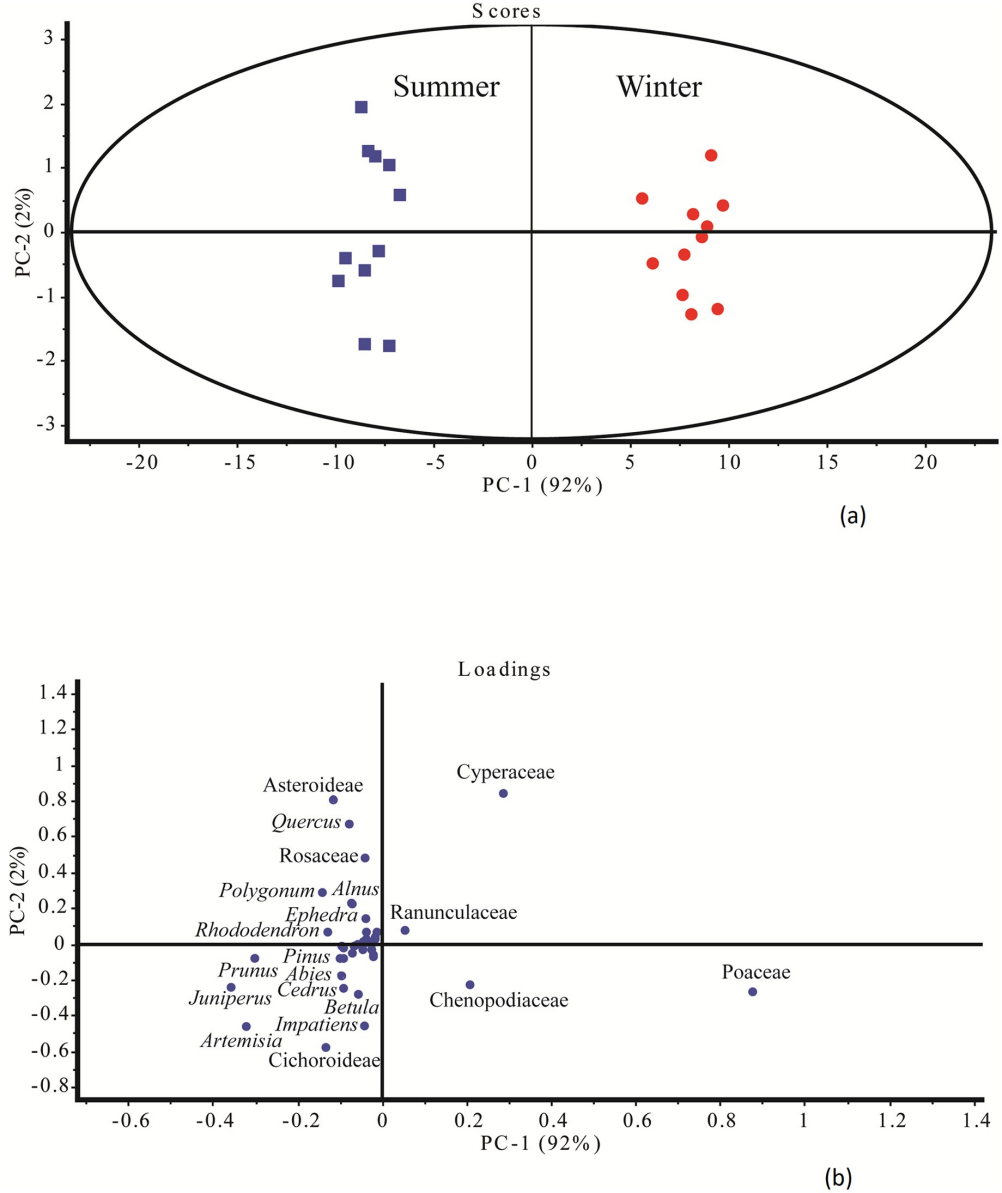
ThePCA analysis of the Yak dung between summer (a) and winter (b) samples.

A total of 94% variance could be explained by two major pollen groups, arboreal and herbaceous taxa (Fig 6a). The score plot showed that these two major components were responsible for the cluster differentiation. Poaceae, Cyperaceae, Chenopodiaceae, and Asteroideae are dominant and placed a high range of the PCA quadrants. The multivariate PCA and the loading plot between PC-I vs PC-II based on the differential pollen frequencies showed different pollen types responsible for cluster separation. The loading plot showed that the pollen taxa responsible for the difference between the summer and winter dung samples are Asteroideae, *Quercus*, Rosaceae, *Polygonum*, *Alnus*, *Ephedra*, *Rhododendron*, *Pinus*, *Prunus*, *Abies*,*Cedrus*, *Betula*, *Impatiens*, *Artemisia*, and Cichoroideae (higher in summer dung). Whereas, taxa like Poaceae, Cyperaceae, Ranunculaceae, and Chenopodiaceae are higher in winter dung samples (Fig 6b).

### Macrobotanical assemblage

**Fig 7:**
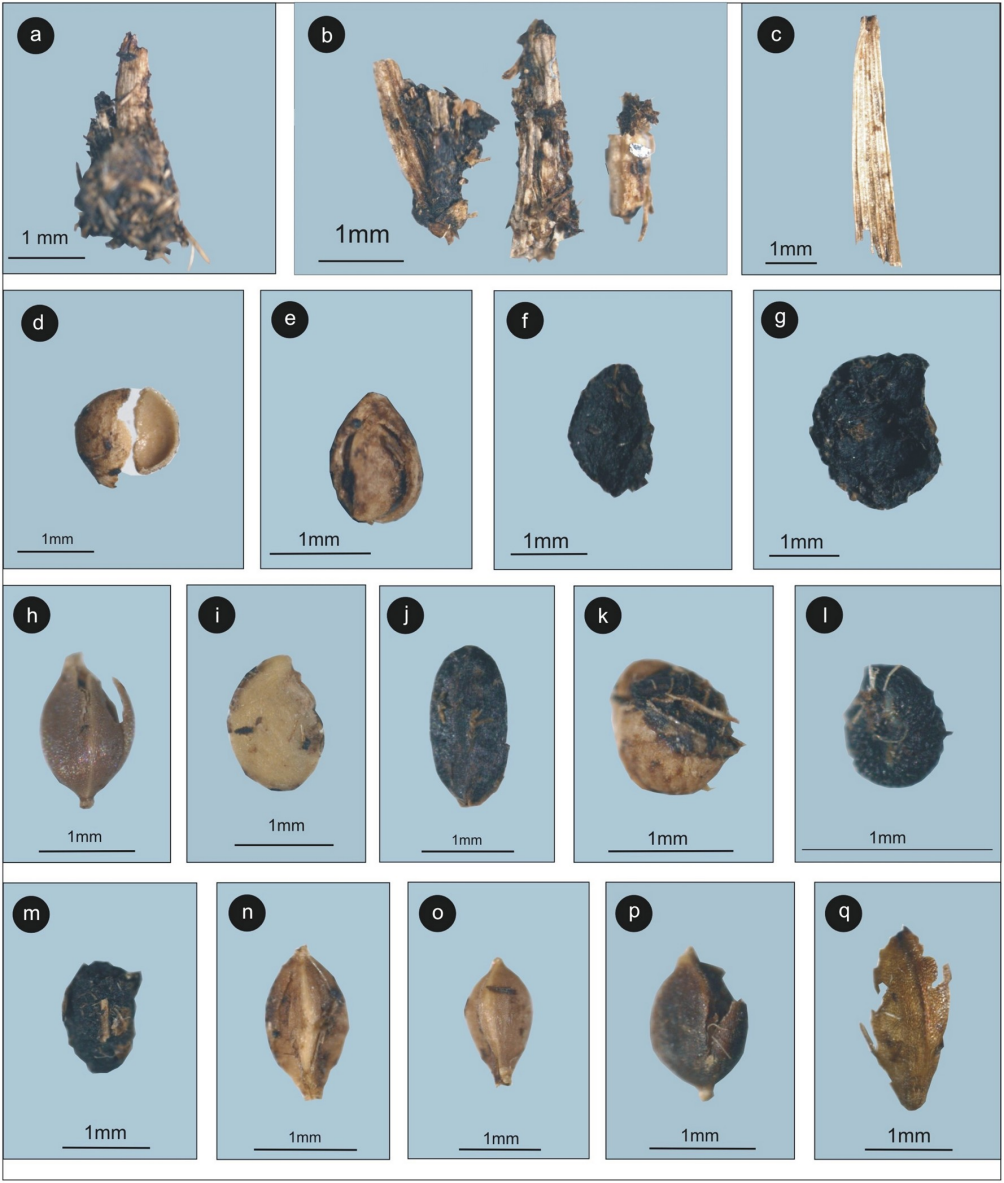
Macrobotanical assemblage recovered from the Yak dung samples. a. & b. plants parts including roots and stem of sedges/herbaceous species, c. Monocot leaf, d. & e. Rosaceae, f. Liliaceae, g. *Xanthoxylum* sp., h. *Polygonum*, i. *Solanum* sp. (Solanaceae), j. Cyperaceae, k. Papaveraceae, l. *Agrostema* sp., m. Fabaceae, n. & o. *Carex* sp. (Cyperaceae), p. Polygonaceae, q. Cyperaceae.

The macrobotanical remains recovered from both summer and winter dung samples are well-preserved. Twigs and leaves of Poaceae were predominant over other herbaceous plant material from the studied samples. Monocot twigs, leaves, and dicotyledonous seeds were also present and may have become incidentally ingested along with the preferred food material (Fig 7).

### FESEM-EDS data

**Fig 8:**
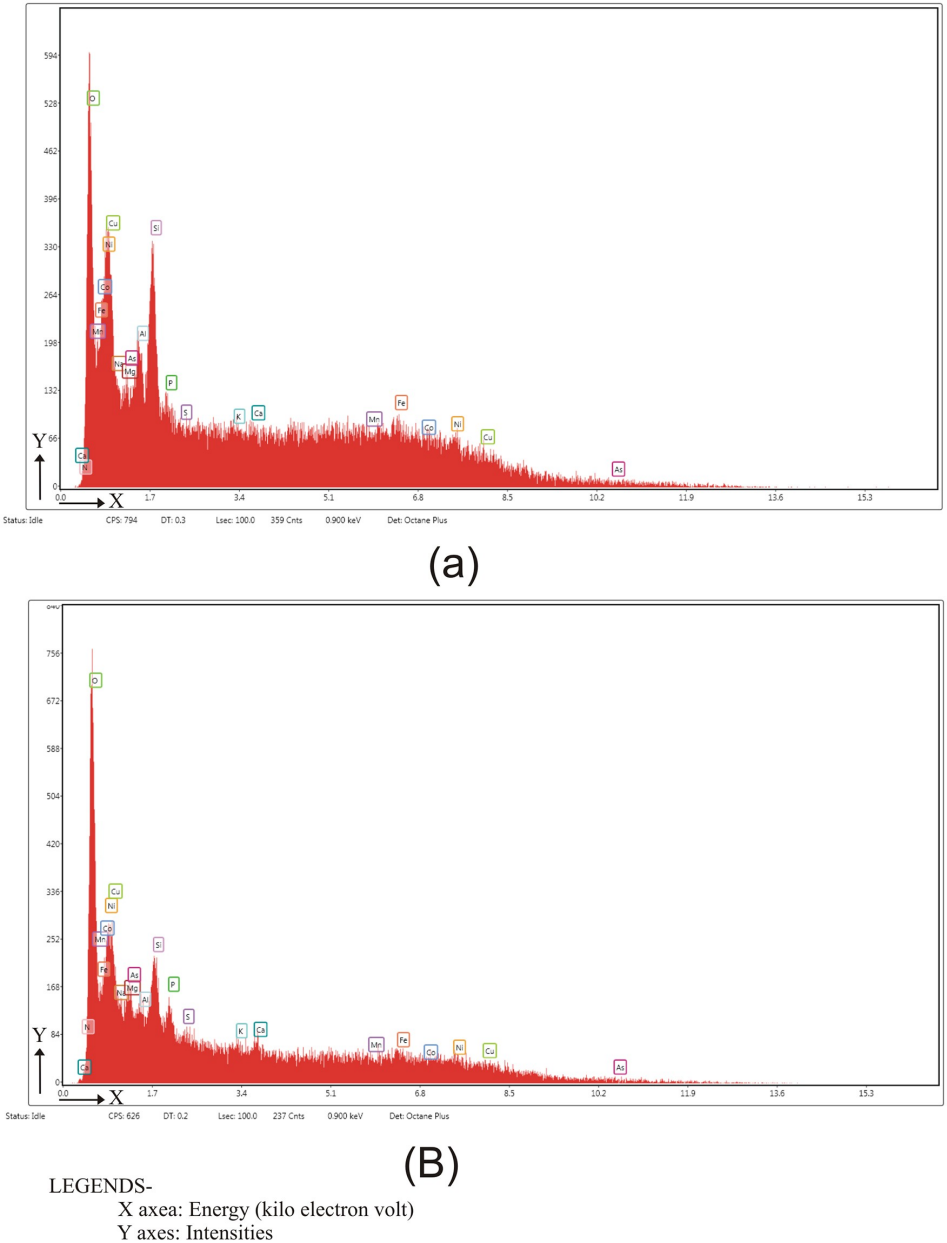
FESEM-EDS analysis micrographs in Yak dung samples collected from summer (a) and winter (b).

The data generated from the FESEM-EDS elemental analysis of the summer yak dung samples observed that the Oxygen (O_2_) content/level is 56.89 (weight %), followed by Na, 18.95 (weight %), Si, 6.4 (weight %), Al, 4.12 (weight %), and Mg, 3.91 (weight %) Fig 8a, (Table 1).

**Table 1:** List of the elements value generated by FESEM-EDS analysis in Yak dung sample collected from summer season.

Element	Weight %	Atomic %	Error %	Net Int.	K Ratio	Z	R	A	F
N K	0.04	0.05	99.99	0.01	0.0001	1.0631	0.9691	0.241	1
O K	56.89	68.79	8.79	64.81	0.2416	1.0436	0.9792	0.4069	1
Na K	18.95	15.95	14.88	22.41	0.0493	0.9527	1.0053	0.2726	1.0029
Mg K	3.91	3.11	18.28	6.56	0.0111	0.9703	1.0128	0.2904	1.0044
Al K	4.12	2.96	15.24	9.27	0.0151	0.9355	1.02	0.3883	1.006
Si K	6.4	4.41	10.22	18.75	0.0304	0.9571	1.0268	0.4931	1.006
P K	3.03	1.89	13.42	8.41	0.016	0.9202	1.0332	0.5685	1.0072
S K	1.54	0.93	12.29	5.08	0.0096	0.9392	1.0393	0.6605	1.0089
K K	1.16	0.57	19.16	4.01	0.0094	0.8915	1.0559	0.887	1.0238
Ca K	0.84	0.41	31.05	2.63	0.0073	0.9086	1.0608	0.9227	1.0283
Mn K	0.02	0.01	99.99	0.05	0.0002	0.8016	1.0816	1.0071	1.1281
Fe K	0.06	0.02	81.78	0.12	0.0006	0.8151	1.0848	1.0118	1.1614
Co K	0.03	0.01	92.07	0.06	0.0003	0.7973	1.0875	1.0146	1.2068
Ni K	0.15	0.05	68.23	0.25	0.0015	0.8252	1.0898	1.0162	1.202
Cu K	2.42	0.74	21.75	3.38	0.023	0.7844	1.0915	1.0168	1.1892
As K	0.45	0.12	67.31	0.31	0.0047	0.7355	1.0907	1.0133	1.391

The FESEM-EDS elemental analysis of the winter yak dung samples recorded that the Oxygen (O_2_) content/level is 33.82 (weight %), followed by Mn, 12.27 (weight %), Ni, 11.47 (weight %), Co, 11.12 (weight %), Fe, 10.9 (weight %), Cu, 8.76 (weight %), and Si, 8.42 (weight %) Fig 8b, (Table 2).

**Table 2:** List of the elements value generated by FESEM-EDS analysis in Yak dung sample collected from winter season.

Element	Weight %	Atomic %	Error %	Net Int.	K Ratio	Z	R	A	F
N K	0.04	0.07	99.99	0.01	0.0001	1.1582	0.9016	0.2556	1
O K	33.82	61.84	12.31	34.38	0.1513	1.1388	0.9129	0.3928	1
Na K	0.02	0.03	99.99	0.01	0	1.0439	0.9428	0.1259	1.0017
Mg K	0.01	0.01	99.99	0.01	0	1.0644	0.9518	0.1987	1.003
Al K	0.32	0.35	86.52	0.5	0.001	1.0273	0.9603	0.2906	1.0054
Si K	8.42	8.77	15.33	18.98	0.0363	1.0521	0.9684	0.4075	1.006
P K	0	0	99.99	0.01	0	1.0125	0.9762	0.4766	1.0099
S K	0	0	99.99	0	0	1.0343	0.9836	0.5938	1.0152
K K	0.19	0.14	66.28	0.59	0.0016	0.9841	1.0042	0.8487	1.0523
Ca K	0.71	0.52	56.3	2.1	0.0068	1.0037	1.0105	0.8968	1.0727
Mn K	12.27	6.53	7.82	25.14	0.125	0.8887	1.0381	0.9967	1.1507
Fe K	10.9	5.71	8.44	19.65	0.1098	0.9044	1.0427	0.9996	1.1141
Co K	11.12	5.52	10.06	16.57	0.104	0.8853	1.047	0.9763	1.0826
Ni K	11.47	5.72	9.31	15.27	0.1068	0.917	1.0508	0.9624	1.0547
Cu K	8.76	4.03	14	9.62	0.0771	0.8725	1.0542	0.9529	1.0592
As K	1.95	0.76	59.62	0.98	0.0174	0.8216	1.0612	0.9687	1.121

## Discussion

The micro- and macrobotanical remains preserved in the summer and winter yak dung, and their elemental analysis indicates seasonal differences in the yak’s dietary preference in relation to the vegetation composition and climate in the region. The overall pollen data of both the summer and winter collected yak dung samples indicate that grasses form the primary component of their diet. The other associated herbs and shrubs, Cyperaceae, *Artemisia*, Asteroideae, Chenopodiaceae, *Impatiens*, *Prunus*, and *Rhododendron* are also very important parts of the species diet. It is also observed that yak prefer diverse plants in their diet in summer as demonstrated by the higher pollen diversity in the summer samples and Yak migrate up to 50 kilometres to forage for their food [40,41].

The presences of Cyperaceae, Rosaceae, Ranunculaceae, and Polygonaceae in the palynoassemblage are also identified in summer and winter seasons and are characteristic of Alpine meadow vegetation in the region. The grasses recovered are closely associated with Rosaceae, Fabaceae, Asteraceae, and Lamiaceae which dominate in Alpine meadow vegetation regarding cover and abundance. Alpine steppes are characterized by high percentages of Chenopodiaceae, and *Artemisia* along with *Ephedra* and *Nitraria* [42]. The association of *Artemisia*, *Caraguna*, *Ephedra*, *Juniperus*, *Salix*, and *Lonicera* is characteristic of the Alpine steppe vegetation in the higher portions of the Himalaya [43]. As our studied samples also included *Ephedra*, *Juniperus*, Chenopodiaceae, and *Artemisia* pollen in the palynoassemblage and complements the presence of Alpine steppe vegetation in and around the studied region. The presence of *Ranunculus* is noticeable in the studied palynoassemblage and found to be characteristic taxa of snow bed vegetation [44]. The presence of Cyperaceae, Polygonaceae, and Onagraceae along with *Botryococcus*, *Hantzschia*, and *Arcella* seen in the dung samples are indicative of the perennial water-logged condition and streamlets running in and around the studied areas. Their absence in the winter samples reflects the absence of unfrozen water. The recovery of shrubby taxa, *Rhododendron* and *Prunus*, along with *Impatiens* and Euphorbiaceae in the palynoassemblage is strongly suggestive of the monsoonal activity in the region [3,45]. The recovery of Chenopodiaceae and *Artemisia* is very significant as these taxa are strongly indicative of the winter dryness in the region. The abundance of *Artemisia* pollen in the winter palynoassemblage indicates the seasonal glacial condition/phase in the region [46] which is reflected in our studied samples. The relative dryness/wetness in the Alpine steppes and deserts could be determined through the ratio of Chenopodiaceae/*Artemisia* pollen in surface soil [42, 47–50]. However, due to presence of Chenopodiaceae and *Artemisia* in dung samples, this ratio could be studied for the climatic interpretation.

Among arboreal taxa, the presence of *Pinus*, *Cedrus*, and *Alnus* in the palynoassemblage which do not grow in this region is indicative of upthermic wind activity which transported the pollen grains from the conifer forest zone present at lower elevations of 1000 to 3300 meters. Pollen from these taxa in the dung samples would have been incorporated through secondary ingestion of the food plant, exposed dung, and soils in the region on which they would have settled. Likewise, the low value of *Juniperus* pollen would have also been incorporated through the ingestion of the plants. *Rhododendron* pollen is entomophilous, its presence in the assemblage would have been incorporated through the ingestion of the plant’s flowers and must have been local in origin. The presence of *Salix* and *Prunus* pollen in the palynoassemblage is indicative of the presence of perennial water channels, streams and moist condition in the region as these plants generally grow along water channels and moist places [42,44,51]. Similarly, the presence of fern spores, both monolete and trilete, in the palynoassemblage is indicative of the warm and humid condition in the region during the summer.

The diatom and phytolith analysis from both summer and winter dung samples revealed that only a few diatom taxa were recovered in the summer dung samples. The presence of diatoms is suggestive of at least seasonal perennial water in the region which became incorporated in the dung through the ingestion of the water. Among phytoliths, grass phytoliths predominant as are the macrobotanical remain of grasses while the other phytoliths also constantly represented in the assemblage and are also indicative of plants other than grasses that were also important food plants of the yak.

The FESEM-EDS elemental analysis of Yak dung samples was also conducted to understand the elemental percentage in relation to the vegetation composition and climate in the region. Sixteen elements have been identified and characterized in the yak dung samples (Tables 1 & 2). As the large animals play an important role in the nutrient cycles due to their ability to travel long distances [29,30], large-bodied forms such as the yak can perform a critical role in this cycle as indicated by the elemental values seen in the dung samples. The redistribution of these elements would play an important role for both plants and animals in the region. For example, the distribution of phosphorous and sodium have been identified as having an important role in the extinction of the both plants and animals in the region [20]. These elements in the yak dung samples could be useful to understand the nutritional value in relation to the vegetation composition and the species dietary preferences in the region.

The macrobotanical assemblage (Cyperaceae, Polygonaceae, and Rosaceae) is in good agreement with pollen data in dung samples in relation to the current vegetation, and climate in the region (Fig 7).

### Biodiversity of summer and winter dung samples

There are some differences between the summer and winter dung samples in the palynoassemblages. The pollen diversity in summer samples is comparatively higher than the winter samples. In summer, the forage is relatively abundant and nutritious and the yak move up to higher altitudes and occupy a wider area including both Alpine meadow and steppe vegetation regions (Fig 1h). During the winter season, they may either moves towards lower elevations or remain in the higher mountain sides with minimal movement reflecting the scarcity of forage due to snow cover at high altitude. Most of the plant taxa, especially arboreal blooms in the summer season and therefore the diversity of pollen taxa in summer dung samples is always higher than winter dung samples. The presence of *Botryococcus*, *Arcella*, and *Hantzschia* in the summer samples is higher than in the winter samples reflecting the greater availability of free-flowing water. In the elemental analysis, the silica content is comparatively higher in the winter samples in response to the dominance of grass pollen.

It is observed that the elemental contents of summer samples were different than the winter samples. The Na, Mg, and Al content is low in the winter samples, and the reason may be due to the scarcity of food plants. Phosphorous and sulfur is absent in the winter samples. However, the O, Mn, Ni, Co, Cu, and Si content in the winter sample is relatively higher than the summer sample. The diversity of the seeds, fruits and twigs is comparatively higher in the winter samples as the maximum occurrences of flowering, and fruiting and the twig of the shrubby elements are more due to being consumed by the yak during that time.

### Statistical significance of pollen frequencies

A critical review of the PCA indicates a clear seasonal periodicity in the appearance of the pollen of different plant species especially the herbaceous taxa, which are in full bloom during the winter season. A clear-cut seasonal clustering of these pollen taxa is present and mainly represented by two major pollen groups, arboreals and herbs. The major arboreal plants like *Pinus*, *Abies*, *Cedrus*, *Betula*, *Quercus*, *Rhododendron*, and *Prunus* are recorded in the summer samples of yak dung, attributed to their maximum blooming in spring season (February to March) and further deposition on herbaceous vegetation, and could be secondarily incorporated as yak’s diet. The dominance of Poaceae, Ranunculaceae, and Chenopodiaceae in winter dung could coincide with their peak of flowering during the winter season. It is also clear from the loading plot that maximum diversity of plant taxa in yak dung occurs in summer season owing to their activities in summer where they cover a larger range. However, in winter during snow fall, they tend to be more inactive finding place for their sustenance, with a dependence on primary herbaceous plant taxa (Fig 6).

### Linkages with endangered and extinct megaherbivores

A study conducted on stomach contents including pollen and spores from fossil woolly rhinoceros (*Coelodonta antiquitatis*) from Russia [16]revealed the presence of predominately non-arboreal pollen taxa including Poaceae and other associated herbs (98.5%), followed by arboreal pollen (trees and shrubs) (0.9%) and spores (including ferns and mosses) (0.6%) respectively. Our dataset for the pollen assemblage from the yak dung samples is closely similar to that of the woolly rhino stomach contents pollen fossil data. Similarly, a study conducted on the mammoth diet based on dung indicates a similarity between these two megaherbivorous mammals, with Poaceae in both taxa as the primary component of their diet followed by the Cyperaceae and Asteraceae. The absence or only trace amounts of arboreal pollen taxa is indicative of the grassland vegetation utilized by the mammoth which lived in cold climatic conditions at higher latitudes [44], that were comparable to the vegetation of the higher elevations at which the yak lives today.

It should be noted that fossil yak has been found from the Pleistocene of eastern Russia, Tibet, and Nepal [52] and so was directly associated with woolly mammoths, and most likely also the woolly rhino as shown by the similar pollen assemblage in the yak dung presented here and coprolite of the woolly mammoths and woolly rhino [16,44]. The woolly rhinoceros and mammoth became extinct in Eurasia because of landscape changes during Pleistocene-Holocene boundary (12000-9000years BP) due to the formation of widespread forest in the temperate and arctic regions of northern Eurasia and loss of grassland [53]. Yak and Bison share a common ancestry and both are considered to originate in central Asia, and mitochondrial DNA analysis also suggests that the yak is closely related to bison [54,55], although they are disjunctly distributed. While the yak remained restricted to western Asia, bison dispersed westward into Europe and northeast across the Bering Land Bridge into North America during the middle to late Pleistocene [50–52, 56–59]. Fossil yak has been reported from Alaska, but radiocarbon dates of these specimens have shown that they are of domestic cattle brought in by miners [60]. The extinct bison (*Bison priscus*) is associated with yak in Eurasian faunas. Studies of its diet based on microhistological fecal analysis indicate that like the mammoth and woolly rhinoceros 98% of its diet consists of grasses, followed by Cyperaceae [61]. However, unlike these faunas, the diet of bison changed between the late Pleistocene and the early Holocene which may have permitted the survival of the recent bison (*Bison bison*) in North America and the wisent (*Bison bonasus*) in Europe [62].

This data are helpful for understanding the extinction of megaherbivorous animals such as woolly rhino and mammoth despite having diets very similar to the surviving yak and bison based on their similar palynodata. There are differences between the woolly rhino and today’s living one horn rhino regarding habit and diet. A study of the pollen and non-pollen palynomorphs preservation in the dung of the extant one horn rhino [3,63] reveals that while grass is the primary diet of both species, the living rhino also required perennial water-logged conditions and a flood plain area that included both large grasslands along with scattered woodland.

## Conclusions

The multiproxy data presented here suggest that the yak utilizes a combination of both Alpine meadow and steppe vegetation depending on the season. Its response to the seasonally cool climate is either its migration to lower elevations or minimizing movements at higher elevations owing to scarcity of food plants. The presence of marshy taxa and diatom in yak’s dung suggests the existence of perennial water system in the study and vicinity areas. So, this documented data might serve as a strong proxy to interpret vegetation and climatic shifts in the higher Himalaya and to correlate them at a global level. Climate change with higher average temperatures may extend the length of time that unfrozen water may be available throughout the region, providing the yak with an opportunity to extend its range. However, as a species adapted to cool temperatures, any increase in mean summer temperatures may force the species to spend more time at higher elevations, above the lusher Alpine meadows, thus reducing access to a major source of food, and also reducing its overall range.

The dung of herbivorous mammals can provide a durable substrate that allows the investigation of the modern pollen data that complements the data recovered from modern surface soil in relation to the vegetation composition and climate in the region. The study of macrobotanical remains, diatoms, and phytoliths can be used to supplement the pollen database to prevent incorrect interpretation for the palaeodietary analysis, when wind transported pollen such as *Pinus*, *Cedrus*, and *Abies* are present in the assemblage. The elemental analysis of different elements in the dung sample also provides a better understanding of the relationship between the surface soil samples and vegetation composition in the region. This multiproxy dataset can help to understand the collapse of species and the subsequent extinction of the megafauna from the different region of the world. The generated data will be helpful for the differentiation of the temperate and tropical megaherbivorous animals in relation to the database. The diet of wild Yak existing in the western Himalayas also includes the consistent occurrence of some secondary herbs and trees beside, grasses as primary food, thus this flexibility in dietary habit could be one of the reason for their survival through Pleistocene-Holocene vegetation transition where other megafaunas become extinct.

While it is clear that, yak, bison, rhinos and mammoth, are capable of feeding on the same types of vegetation, predominately grasses, and so had similar preferences in their diet, there were significant differences in their preferred habitat. The wild yak survives today, although with a much-reduced distribution. Its current distribution corresponds to the combination of the vegetation composition and colder climate that exists at the higher elevations in the Himalayan region. Possibly due to their larger size and need for greater quantities of food the larger mammoth and rhino were not able to make the transition to, this habitat. The closest living relative to the woolly rhino is *Dicerorhinus sumatrensis*, with the genus *Rhinoceros* (*unicornis* and *sondaicus*) forming their sister group [64]. This raises an interesting paradox since the high elevations of the Tibetan Plateau has been proposed as the area of origin of the woolly rhino [65]. In contrast the bison survived by a change in its diet. So, while this diversity of grazers all lived at the same time and shared a common habitat, they reflect the three ways to respond to climate change; track the preferred habitat, adapt to changing conditions or go extinct.

## Supporting information

S1 Table Pollen frequencies data generated from Yak (*Bos mutus*) dung samples. (XLSX)

## References

[1] Carrion J. A taphonomic study of modern pollen assemblages from dung and surface sediments in arid environments of Spain. Review of Palaeobotany and Palynology. 2002; 120:217–232.

[2] Kropf M, Mead JI, Anderson RS. Dung, diet, and the paleoenvironment of the extinct shrub-ox (*Euceratheriumcollinum*) on the Colorado Plateau, USA. Quaternary Research. 2007; 67:143–151.

[3] Basumatary SK, McDonald HG, Gogoi Rajib. Pollen and non-pollen palynomorph preservation in the dung of the Greater One-horned Rhino (*Rhinoceros unicornis*), and its implication to palaeoecology and palaeodietary analysis: A case study from India. Review of Palaeobotany and Palynology. 2017; 244:153–162.

[4] Bent AM, Wright HE. Pollen analyses of surface materials and lake sediments from the Chiska Mountains, New Mexico. Geological Society of America Bulletin. 1963; 74:491–500.

[5] Janssen CR. A comparison between the recent regional pollen rain and the subrecent vegetation in four major vegetation types in Minnesota (U.S.A.). Review of Palaeobotany and Palynology. 1967; 2: 331–342.

[6] Prentice IC. Pollen representation, source area and basin size: toward a unified theory of pollen analysis. Quaternary Research. 1985; 23:76–86.

[7] Xu Q H, Li YC, Yang XL, Xiao JL, Liang WD, Peng YJ. Source and distribution of pollen in the surface sediment of Daihai lake, inner Mongolia. Quaternary International. 2005; 136:33–45.

[8] Gosling WD, Mayle FE, Tate NJ, Killeen TJ. Differentiation between Neotropical rainforest, dry forest, and savannah ecosystems by their modern pollen spectra and implications for the fossil pollen record. Review of Palaeobotany and Palynology. 2009; 153:70–85.

[9] Quamar MF, Ali SN, Pandita SK, Singh Y. Modern pollen rain from Udhampur (Jammu and Kashmir), India: Insights into pollen production, dispersal, transport and preservation. *Palynology*. 2017; 1–11. 10.1080/01916122.2017.1306811

[10] Bajpai R, Kar R. Modern pollen deposition in glacial settings in the Himalaya (India): abundance of *Pinus* pollen and its significance. Palynology, 2018; 10.1080/01916122.2017.1407835

[11] Moe D. Palynology of Sheep’s faeces: relationship between pollen content, diet and local pollen rain. Grana. 1983; 22:105–113.

[12] Galloway JM, Adamczewski J, Schock DM, Andrews TD, MacKay G, Bowyer VE, et al Diet and Habitat of Mountain Woodland Caribou Inferred from Dung Preserved in 5000-year-old Alpine Ice in the Selwyn Mountains, Northwest Territories, Canada. Arctic. 2012; 6:59–79.

[13] Martin PS, Sharrock FW. Pollen analysis of prehistoric human feces: a new approach to ethnobotany. Am. Antiq. 1964; 30:168–180.

[14] Hall HJ. Diet and Disease at Clyde’s Cavern, Utah: As Revealed via Paleoscatology. Unpublished M.A. Thesis University of Utah, Salt Lake City, 1972.

[15] Schoenwetter J. Pollen analysis of prehistoric human feces from Salts Cave, Kentucky In: Watson P. (Ed.), Archeology of the Mammoth Cave Area. Academic Press, New York, USA, pp. 203–209; 1974.

[16] Boeskorov GG, Bakulina NT, Davydov SP, Shchelchkova MV, Solomonov NG. Study of Pollen and Spores from the Stomach of a Fossil Woolly Rhinoceros Found in the Lower Reaches of the Kolyma River. Doklady Biological Sciences. 2011; 436:23–25. 10.1134/S0012496611010017 21374006

[17] Gravendeel B, Protopopov A, Bull I, Duijm E, Gill F, Nieman A, et al Multiproxy study of the last meal of a mid-Holocene OyogosYar horse, Sakha Republic, Russia. The Holocene. 2014; 24:1288–1296.

[18] Langeveld BV, Mol D, Zazula GD, Gravendeel B, Eurlings M, McMichael CNH. et al, A multidisciplinary study of a Late Pleistocene arctic ground squirrel (*Urocitellusparryii*) midden from Yukon, Canada. Quaternary Research. 2018; 89:333–351.

[19] Knapp A K, Blair JM, Briggs JM, Collins SL, Hartnett DC, Johnson LC et al The keystone role of bison in North American tall grass prairie. Bioscience. 1999; 49:39–50.

[20] Doughty CE, Wolf A, Malhi Y. The legacy of the Pleistocene megafauna extinctions on nutrient availability in Amazonia. Nature Geoscience; 2013; 6:761–764.

[21] Giombini MI, Bravo SP, Tosto DS. The key role of the largest extant neotropicalfrugivore (Tapirusterrestris) in promoting admixture of plant genotypes across the landscape. Biotropica. 2016; 48:499–508.

[22] Sridhara S, McConkey K, Prasad S, Corlett RT. Frugivory and seed dispersal by large herbivores of Asia In: Ahrestani F.S., Sankaran M. (Eds.), The Ecology of Large Herbivores in South and Southeast Asia. Springer, Dordrecht, the Netherlands, pp. 121–150; 2016.

[23] Graves P, Kealhofer L. Assessing bioturbation in archaeological sediments using soil morphology and phytolith analysis. Journal of Archaeological Science. 1999;26:1239–48.

[24] Lu H, Liu K. Phytoliths of common grasses in the coastal environments of southeastern USA. Estuarine, Coastal and Shelf Science. 2003; 58:587–600.

[25] Gallego L, Distel RA. Phytolith assemblages in grasses native to central Argentina. Annals of Botany; 2004; 94:865–874. 10.1093/aob/mch214 15514027 PMC4242281

[26] Blinnikov MS. Phytoliths in plants and soils of the interior Pacific Northwest, USA. Review of Palaeobotany and Palynology.2005; 135:71–98.

[27] Lu H, Wu N, Yang X, Jiang H, Liu K, Liu T. Phytoliths as quantitative indicators for the reconstruction of past environmental conditions in China I: phytolith; 2005.

[28] Blinnikov MS, Bagent CM, Reyerson PE. Phytolith assemblages and opal concentrations from modern soils differentiate temperate grasslands of controlled composition on experimental plots at Cedar Creek, Minnesota. Quaternary International, 2013; 287:101–113.

[29] Kelt DA, Van Vuren DH. The ecology and macroecology of mammalian home range area. Am Nat. 2001; 157:637–645. 10.1086/320621 18707280

[30] Demment MW, Van Soest PJ. A Nutritional Explanation for Body-Size Patterns of Ruminant and Nonruminant Herbivores. Am Nat. 1985; 125:641–672.

[31] Chundawat RS. Rawat GS. Indian cold desert: A status report on biodiversity, Wildlife Institute of India, Dehradun; 1994.

[32] Lu ZL. Reproduction and conservation of wild yak. In: Zhao, X.X., Zhang, R.C. (Eds.), Recent Advances in Yak Reproduction. International Veterinary Information Services (www.ivis.org), Ithaca, New York, 2000.

[33] Schaller GB, Kang A, Hashi TD, Cal P. A winter wildlife survey in the northern Qiangtang of Tibet Autonomous Region and Qinghai Province, china. Acta theriologicasinica. 2007; 27: 309–316.

[34] Berger J, Cheng E, Kang A, Krebs M, Li L, Lu ZX. et al Sex difference in ecology of wild yaks at high elevation in the Kekexili reserve, Tibetan Qinghai Plateau, China. Journal of Mammology. 2014; 95(3): 638–645. 35.

[35] Han J. Wild yak (*Bos mutus*Przewalski, 1883) Part III, Chapter-12: Cambridge University Press; pp 204; 2014.

[36] Erdtman G. An Introduction to Pollen Analysis. Waltham, Mass, USA; 1953.

[37] Gupta HP, Sharma C. Pollen flora of North-west Himalaya. Indian Association of Palynostratigraphers, Lucknow, India, 1986; pp. 3–142.

[38] Farooqui A, Gaurm AS, Prasad V. Climate, vegetation and ecology during Harappan period: excavations at Kanjetar and Kaj, Mid-Saurashtra coast, Gujarat. Journal of Archaeological Science. 2013; 40:2631–2647.

[39] Madella M, Alexandre A, Ball T. International code for Phytolith Nomenclature. Annals of Botany. 2005; 96:253–260. 10.1093/aob/mci172 15944178 PMC4246872

[40] Zhao JN, Gao XY. The wild yak. Arid Zone Research 1991; 1: 34. (In Chinese).

[41] Yao J, Yang BH, Yan P, Liang CN, Guo J, Jiao S. Analysis on habitat variance and behaviours of *Bos grunniens* in China. Acta PrataculturaeSinica 2006; 15:124–128. (In Chinese with English abstract).

[42] Yu G, Tang LY, Yang XD, Ke XK, Harrison SP. Modern pollen samples from alpine from alpine vegetation on the Tibetan Plateau. Global Ecology & Biogeography. 2001; 10: 503–509.

[43] Manjrekar N. Feeding Ecology of Ibex (Capra ibex sibirica) in Pin Valley National Park, Himachal Pradesh. PhD Thesis. Saurashtra University, Rajkot, India; 1998.

[44] van Geel B, Aptroot A, Baittinger C, Birks HH, Bull ID, Cross HB, et al The ecological implications of a Yakutian mammoth’s last meal. Quaternary Research. 2008;69:361–376.

[45] Swati Dixit, Bera SK. Holocene climatic fluctuation from lower Brahmaputra floodplain of Assam, Northeast India. Journal of Earth System Science; 2012;121:135–147.

[46] Subally D, Quezel P. Glacial or interglacial: Artemisia, a plant indicator with dual responses. Review of Palaeobotany and Palynology. 2002; 120:123–130.

[47] El-Moslimany AP. Ecological significance of common non-arboreal pollen: examples for drylands of the Middle East. Review of Palaeobotany and Palynology. 1990; 64:343–350.

[48] Cour P, Zheng Z, Duzer D, Calleja M, Yao Z. Vegetational and climatic significance of modern pollen rain in northwestern Tibet. Review of Palaeobotany and Palynology. 1999; 104:183–204.

[49] Herzschuh U. Reliability of pollen ratios for environmental reconstructions on the Tibetan Plateau. Journal of Biogeography. 2007; 34:1265–1273.

[50] Zhao Y, Liu H, Li F, Huang X, Sun J, Zhao W, et al Application and limitations of the *Artemisia*/Chenopodiaceae pollen ratio in arid and semi-arid China. The Holocene. 2012; 22:1385–1392.

[51] Srivatava SK, Shukla AN. Flora of cold desert, western Himalaya, India. Botanical Survey of India, Volume 2.PP. 1–5; 2015.

[52] Olsen SJ. Fossil ancestry of the yak, its cultural significance and domestication in Tibet. Proceedings of the Academy of Natural Sciences of Philadelphia. 1990; 142:73–100.

[53] Kuzmin YV. Extinction of the woolly mammoth (*Mammuthusprimigenius*) and woolly rhinoceros (*Coelodontaantiquitatis*) in Eurasia: Review of chronological and environmental issues. Boreas. 2010; 39:247–261.

[54] Guo SC, Liu JC, Qi DL, Yang J, Zhao XQ. Taxonomic placement and origin of yaks: implications from analyses of mtDNA D-loop fragment sequences. Acta TheriologicaSinica. 2006; 26: 325–330.

[55] Miyamato MM, Tanhauser SM, Laipis PJ. Systematic relationships in the artiodactyl tribe Bovini (family Bovidae), as determined from mitochondrial DNA sequences. Systematic Zoology. 1989; 38:342–349.

[56] Pilgrim GE. The fossil Bovidae of India. Memoirs of the Geological Survey of India, New Series 26; 1939; 1–356 + plates I–VIII.

[57] Groves CP. Systematic relationships in the Bovini (Artiodactyla, Bovidae). Zeitschrift fur ZoologischeSystematik Und Evolutionsforschung. 1981; 19:264–278.

[58] McDonald JN. North American bison: their classification and evolution. University of California Press, Berkeley; 1981.

[59] Meagher M. Bison bison. Mammalian Species. 1986; 266:1–8.

[60] Guthrie RD. Frozen Fauna of the Mammoth Steppe, The Story of Blue Babe. University of Chicago Press, Chicago; 1990.

[61] Sparks DR, Malechek JC. Estimating percentage dry weight in diets using a microscopic technique. Journal of Range Management. 1968; 21:264–265.

[62] Rivals F, Solounias N, Mihlbachler MC. Evidence for geographic variation in the diets of late Pleistocene and early Holocene Bison in North America, and differences from the diets of recent Bison. Quaternary Research. 2007; 68:338–346.

[63] Basumatary SK, McDonald HG. Coprophilous fungi from dung of the Greater One-Horned Rhino in Kaziranga National Park, India and its implication to paleoherbivory and paleoecology. Quaternary Research. 2017; 88:14–22.

[64] Orlando L, Leonard JA, Thenot V, Laudet C, Guerin, Hänni C. Ancient DNA analysis reveals woolly rhino evolutionary relationships. Molecular Phylogenetics and Evolution. 2003; 28:485–499. 12927133 10.1016/s1055-7903(03)00023-x

[65] Deng T, Wang X, Fortelius M, Li Q, Wang Y, Tseng ZJ, et al Out of Tibet: Pliocene Woolly rhino suggests Highhplateau origin of Ice Age megaherbivores. Science. 2011; 6047:1285–1288. 10.1126/science.1206594 21885780

